# Weighted set enrichment of gene expression data

**DOI:** 10.1186/1752-0509-7-S4-S10

**Published:** 2013-10-23

**Authors:** Rehman Qureshi, Ahmet Sacan

**Affiliations:** 1 Center for Integrated Bioinformatics, School of Biomedical Engineering, Drexel University, Philadelphia, PA, USA

## Abstract

**Background:**

Sets of genes that are known to be associated with each other can be used to interpret microarray data. This gene set approach to microarray data analysis can illustrate patterns of gene expression which may be more informative than analyzing the expression of individual genes. Various statistical approaches exist for the analysis of gene sets. There are three main classes of these methods: over-representation analysis, functional class scoring, and pathway topology based methods.

**Methods:**

We propose weighted hypergeometric and weighted chi-squared methods in order to assign a rank to the degree to which each gene participates in the enrichment. Each gene is assigned a weight determined by the absolute value of its log fold change, which is then raised to a certain power. The power value can be adjusted as needed. Datasets from the Gene Expression Omnibus are used to test the method. The significantly enriched pathways are validated through searching the literature in order to determine their relevance to the dataset.

**Results:**

Although these methods detect fewer significantly enriched pathways, they can potentially produce more relevant results. Furthermore, we compare the results of different enrichment methods on a set of microarray studies all containing data from various rodent neuropathic pain models.

**Discussion:**

Our method is able to produce more consistent results than other methods when evaluated on similar datasets. It can also potentially detect relevant pathways that are not identified by the standard methods. However, the lack of biological ground truth makes validating the method difficult.

## Introduction

Due to their ability to provide comprehensive snapshots of cellular activity, microarrays have become a widely utilized tool in bio-medical sciences. Microarray-based gene expression detection has been used for biomarker discovery as well as diagnostic and prognostic purposes [1-4]. Online microarray experiment repositories such as Gene Expression Omnibus (GEO) [5,6], ArrayExpress [7], and Stanford Microarray Database (SMD) [8] are invaluable resources containing gene expression profiles that span multiple developmental stages, experimental conditions, and model organisms [9,10]. There are numerous challenges presented by the expanding availability of microarray data. The difficulty of interpreting the lists of significant genes produced by microarray experiments is a major challenge. The staggering number and diversity of the differentially expressed genes can be hard to interpret in a biologically meaningful way. As a result several statistical methods for gene set enrichment have been developed. The set of differentially expressed genes is compared to gene sets from various databases including Gene Ontology (GO) [11] or the Kyoto Encyclopedia of Genes and Genomes (KEGG) [12].

Huang et al. reviewed and classified 68 available tools for the statistical analysis of gene sets [13]. Huang classified the available pathway enrichment methods into three categories: over-representation analysis (ORA), functional class scoring (FCS), and pathway topology (PT) based methods [13,14]. In ORA, a list of genes is compiled by selecting genes based on their significance, fold change, or both. ORA techniques seek to identify whether the gene list is over-represented in a gene set or pathway. In ORA approaches, if *k *genes from the list are found in a pathway then the probability of finding *k *or more genes is calculated. The resulting p-values are used to determine whether or not a pathway or gene set is significantly enriched. The probability can be calculated using the chi-squared distribution, Fisher's Exact Test, the binomial probability distribution, or the hypergeometric distribution [13].

In functional class scoring approaches, such as Gene Set Enrichment Analysis (GSEA) [15], all genes are considered when calculating enrichment instead of a pre-selected list [14,15]. This can deliver improved statistical power [13]. In the FCS approaches, genes are assigned ranks. In GSEA, a gene's rank is determined by its correlation with the experimental sample classifications. When calculating the significance of a gene set, the null hypothesis is that the genes in a set are randomly distributed throughout the ranked list of genes from the microarray experiment. GSEA creates a null distribution by randomly permuting the labels of the samples and producing lists of genes ranked by their correlation with the newly shuffled sample labels. Using this null distribution to estimate the significance is analogous to a weighted Kolmogorov-Smirnov-like statistic [15]. In contrast, Parametric Analysis of Gene Set Enrichment (PAGE) determines a z-score for a set and uses normal distribution to determine significance [16].

Both ORA and FCS approaches ignore the connections between genes in a pathway, however PT-based approaches integrate the information contain in the edges of a pathway when determining the enrichment. The disadvantage of PT-based approaches is that they cannot be applied to the Gene Ontology [14]. ScorePAGE computes a score that represents the similarity between pairs of genes, and then divides this score by the number of edges between the two genes [14,17]. Another approach is that of Signaling Pathway Impact Analysis (SPIA), which computes a "perturbation factor" for each gene in a pathway. This is given by the change in expression of the gene and by a linear function of the perturbation factors of all the other genes in the pathway. The "impact factor" of the pathway is a statistic calculated by taking the sum of the perturbation factors of the genes in the pathway [14,18].

We have previously proposed a method for enriching gene sets that is a hybrid of over-representation and functional class scoring [19]. Our method requires the contribution of all the genes in the dataset. Each gene contributes to the enrichment score in proportion to its fold change. Like ORA we calculate significance using the hypergeometric or chi-square distribution; however our method weighs the probability calculation by the fold change of the genes. In our method each gene is assigned a score based on its fold change, and we create a pseudo pathway, which is proportionally larger than the original pathway. We then calculate the significance of sampling the sum of the scores of the genes from the larger pseudo pathway.

We applied this pathway enrichment methodology in order to perform a meta-analysis of rodent neuropathic pain microarray experiments. Neuropathic pain is a chronic condition resulting from damage to any part of the nervous system or from diseases affecting an area of the nervous system. Neuropathic pain is typically accompanied by inflammation [20] and sensory and motor dysfunction [21]. Up to eight percent of the general population is affected by neuropathic pain [22,23]. While there is no clear etiology for neuropathic pain, spinal cord injury, diabetes, alcoholism, chemotherapy, chronic viral infection, transverse myelitis, and strokes are common causes. Due to the complex etiology and symptoms of neuropathic pain and its poorly understood mechanisms, the classification of chronic pain syndromes has remained largely subjective. Common treatments are able to produce better than moderate pain relief in only one third of patients [24]. Treatments such as opiates, tricyclic antidepressants, anti-convulsants, anti-epileptics, topical analgesics, and NMDA-antagonists are used despite their limited efficacy and harmful side-effects [25,26]. There are several rodent models of neuropathic pain such as nerve ligation, chronic constriction, and spared nerve injury [27,28].

## Methods

### Gene ID mapping

Before we could begin performing enrichment analysis, we needed to construct a back-end database containing relevant information from various databases. Towards this end, we stored all the KEGG pathways and the genes involved in each pathway in a database. We further created a database to map the correspondence of Entrez genes with Affymetrix probe identifiers to enable gene identifier conversion. The correspondence between Entrez gene identifiers and KEGG gene identifiers was also mapped. Ultimately, a database for the KEGG pathway information and a database for gene identifier conversion were created in SQLite. Only genes from *Homo Sapiens, Rattus Norvegicus, and Mus Musculus *were included in the database. We mapped Affymetrix gene identifiers to Entrez Gene identifiers for Affymetrix microarray datasets with binary classifications obtained from the Gene Expression Omnibus (GEO) [6]. Because multiple Affymetrix probes can map to a single Entrez Gene, we took the mean of the fold-change of the corresponding probes and the minimum of their p-values.

### Enrichment

Before calculating enrichment, we quantile-normalized the raw data and computed the fold change of each gene. A two-tailed Student's t-test with an alpha value of 0.01 was used to identify significant differentially expressed genes. The standard hypergeometric test was used to perform enrichment for comparison to our method. The probability of finding *X *>*k *significant genes in a particular gene set or pathway is calculated as follows:

(1) $$P\left(X>k\right)=1-\overset{k}{\underset{r=0}{\sum }}\frac{\left(\begin{array}{c} m\\ r \end{array}\right)\times \left(\begin{array}{c} N-m\\ n-r \end{array}\right)}{\left(\begin{array}{c} N\\ n \end{array}\right)}$$

where *N *is the number of genes on the array, *m *is the number of significant genes, *n *is the number of genes in the particular KEGG pathway, and *k *is the number of genes that are both significant and present in the particular KEGG pathway. Thus we were able to calculate the significance of the enrichment of the KEGG pathways and rank them by their significance.

For our weighted hypergeometric and chi-squared tests, each gene was assigned a score calculated as shown in the formula below:

(2) $$g_{i}={\left|log_{2}\left(fold\;change\left(gene_{i}\right)\right)\right|}^{a}$$

The power, *a*, is an adjustable parameter. In each dataset, a value *Q *was calculated by taking the maximum of the gene scores in the pathway. The hypergeometric distribution is a discrete probability distribution function; however our gene scores existed on a continuous scale. Thus, we had to ensure that our gene scores were discrete. Rounding the values of the gene scores to the nearest whole number accomplished this. For each KEGG pathway, we calculated *k *by taking the sum of the scores of the genes involved in the pathway, as shown in the formula below:

(3) $$k=\overset{n}{\underset{i=1}{\sum }}g_{i}$$

where n is the number of genes in a particular KEGG pathway. Each individual gene's score *g_i_*, corresponded to the number of copies of that gene that were considered significant in the pseudo pathway. The value *k *corresponds to the total number of significant genes in the pseudo pathway. We then utilized the hypergeometric distribution to calculate the probability that the pathway score was greater than *k*, according to the formula below

(4) $$P\left(X>k\right)=1-\overset{k}{\underset{r=0}{\sum }}\frac{\left(\begin{array}{c} N\\ k \end{array}\right)\times \left(\begin{array}{c} QN-N\\ Qn-k \end{array}\right)}{\left(\begin{array}{c} QN\\ Qn \end{array}\right)}$$

where all variables represent the same quantities that they do in equation 1, and all quantities are rounded to the nearest whole number. We ranked the pathways using this p-value.

A similar approach was applied to the chi-squared statistic. The chi-squared distribution represents an approximation of the exact probability of sampling without replacement, which is determined by the hypergeometric distribution. The chi-squared statistic was sometimes used because of the difficulty of computing hypergeometric probabilities for large populations. The chi-squared statistic [29] is determined using the 2x2 table shown in Table 1. The values from Table 1 are used in the equation shown below

**Table 1:** The 2x2 table used to calculate the chi-squared statistic.

	Genes on Array	Significant Genes	
**In Pathway**	n_11_	n_12_	N_1r _= n_11 _+ n_12_

**Not in Pathway**	n_21_	n_22_	N_2r _= n_21 _+ n_22_

	N_1c _= n_11_+n_21_	N_2c _= n_12_+n_22_	N = n_11_+n_12_+n_21_+n_22_

(5) $$\chi^{2}=\frac{N{\left(\left|n_{11}n_{22}-n_{12}n_{21}\right|\right)}^{2}}{N_{1r}N_{2r}N_{1c}N_{2c}}$$

We utilize a chi-squared distribution with 1 degree of freedom, which is calculated from Table 1 as follows:

(6) $$df=\left(r-1\right)\left(c-1\right)$$

where *r *is the number of rows in the table and *c *is the number of columns. We compute a weighted chi-squared statistic by constructing a table similar to Table 1, but in the place of the significant genes column we use the pathway score calculated by Equation 5 and the sum of the scores of all the genes on the array. Unlike the hypergeometric probability distribution, the chi-squared probability distribution is continuous. We did not need to discretize our data.

## Experiments and results

We tested these weighted enrichment approaches using a microarray dataset from that that compares *C. Pneumoniae *infected dendritic cells and mock-infected controls [30]. The authors of the original dataset did not conduct enrichment analysis during their study. The enriched pathways resulting from standard hypergeometric enrichment were compared to the enriched pathways resulting from weighted hypergeometric and chi-squared enrichment. The top-10 most significant pathways detected by hypergeometric enrichment are shown in Table 2. Table 3 shows the top-10 pathways enriched by the weighted hypergeometric method. Table 4 shows the top-10 pathways produced by weighted chi-squared enrichment.

**Table 2:** The results of hypergeometric enrichment of the genes that are significant at the 0

Pathway	p-value	FDR	# of significant genes
Nitrogen metabolism	0.000329	0.080509	4
Biotin metabolism	0.000815	0.099656	1
Prion diseases	0.002291	0.186713	4
Natural killer cell mediated cytotoxicity	0.002489	0.152139	9
Gap junction	0.002548	0.124621	7
Cytokine-cytokine receptor interaction	0.002632	0.107276	15
ErbB signaling pathway	0.002747	0.095982	7
Osteoclast differentiation	0.002976	0.090963	9
Non-small cell lung cancer	0.003207	0.087127	5
Vibrio cholerae infection	0.00428	0.104663	5

**Table 3:** The results of weighted hypergeometric enrichment of the *C.Pneumonia *infection dataset

Pathway	P-Value	FDR	Pathway Score
Glycosphingolipid biosynthesis - globo series	0.011041	1	22
Glycosphingolipid biosynthesis - ganglio series	0.01264	1	23
Glycosaminoglycan degradation	0.019259	1	26
Pantothenate and CoA biosynthesis	0.030492	1	24
D-Arginine and D-ornithine metabolism	0.056692	1	2
Protein export	0.063533	1	26
Vitamin digestion and absorption	0.075501	1	29
Thiamine metabolism	0.101094	1	6
Primary bile acid biosynthesis	0.108834	1	19
Ether lipid metabolism	0.114348	1	40

**Table 4:** The results of weighted chi-squared enrichment of the *C. Pneumonia *infection dataset

Pathway	P-Value	FDR	Pathway Score
Drug metabolism - cytochrome P450	0.034493	1	35.97864
Amyotrophic lateral sclerosis (ALS)	0.076237	1	34.94194
Pathways in cancer	0.081381	1	268.3321
Cell adhesion molecules (CAMs)	0.102061	1	97.04176
Calcium signaling pathway	0.114357	1	140.0101
NOD-like receptor signaling pathway	0.131509	1	39.68646
Glycosphingolipid biosynthesis - ganglio series	0.141836	1	23.47024
Glyoxylate and dicarboxylate metabolism	0.14306	1	9.819872
Glycosphingolipid biosynthesis - globo series	0.145623	1	22.14654
Axon guidance	0.173427	1	102.641

**Figure 1:**
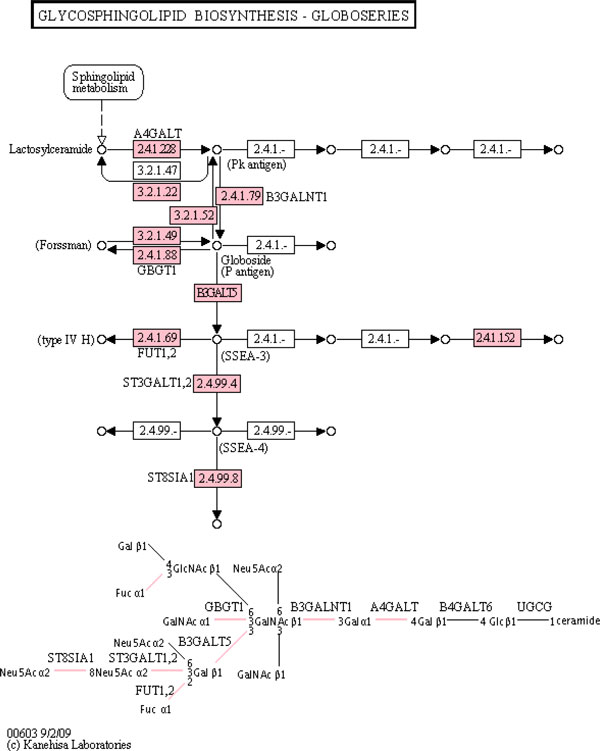
The glycosphingolipid biosynthesis-globo series KEGG pathway, with Entrez Genes detected on the array colored pink; this was the top-ranked pathway by weighted hypergeometric enrichment.

Hypergeometric enrichment detected 56 significant pathways (p < 0.05). Table 3 shows that weighted hypergeometric enrichment only detected four significant pathway (p < 0.05).Weighted chi-squared enrichment only detected one significant pathway, as shown in Table 4. This significant pathway, which contained only 2 significant genes, was ranked 132 by hypergeometric enrichment and had a p-value of 0.23. The two most significant pathways according to weighted hypergeometric enrichment were both glycosphingolipid biosynthesis pathways, which were not detected by the standard method because none of the genes in the pathway were significant despite high fold-change. Despite having no significant genes (p < 0.01), the mean expression change of the genes in the glycosphingolipid biosynthesis--globo series pathway was over 3-fold; specific values for significance and fold-change for the genes in this pathway are shown in Table 5. The glycosphingolipid biosynthesis--globo series pathway [12] is shown in Figure 1. Additionally, there exist both highly upregulated and downregulated genes in the pathway.

**Table 5:** The p-values and fold changes of the genes in the glycosphingolipid biosynthesis-globo pathway

Entrez Gene ID	Symbol	P-Value	Fold Change
2523	*Fut1*	0.154618	1.079052
2524	*FUT2*	0.085096	1.506772
2717	*Gla*	0.048796	7.793774
3073	*HexA*	0.271048	5.073008
3074	*Hexb*	0.166863	7.790576
4668	*nagA*	0.063709	2.171953
6482	*ST3GAL1*	0.275852	2.535963
6483	*ST3GAL2*	0.13051	2.067693
6489	*ST8SIA1*	0.357908	7.755918
8706	*B3galnt1*	0.029243	0.863497
10317	*B3galt5*	0.475799	3.853211
10690	*fut9*	0.275609	1.418501
26301	*Gbgt1*	0.338454	0.097302
53947	*A4GALT*	0.621914	0.619847

We performed a literature search to assess the relevance of the top-10 pathways produced by the various methods. Since the dataset utilized involved the infection of cells, pathways related to the immune system should be enriched. Hypergeometric enrichment identified two potentially relevant pathways: natural killer cell mediated cytotoxicity and *V. Cholerae *infection. The top-2 pathways produced by this method were nitrogen metabolism and biotin metabolism. Weighted hypergeometric enrichment identified both types of glycosphingolipid biosynthesis as the top-2 pathways, with a glycosaminoglycan degradation related pathway as the third ranked pathway. Glycans and glycosylation are essential components of the antigen-presenting function of dendritic cells [31]. Glycosphingolipids are proteins present in the plasma membrane that are known to be involved in immune function. They can act as cell-surface antigens [32,33]. The standard method failed to detect these pathways; where as the weighted hypergeometric method uncovered the action of these pathways and helped elucidate mechanisms of the infection of the cells. In addition, despite finding fewer significant pathways the weighted chi-squared method also detected the glycosphingolipid synthesis pathways among its top-10 pathways, although at lower ranks than weighted hypergeometric enrichment. These pathways, which are the top-ranked pathways by the weighted hypergeometric method, are more biologically relevant than the top-ranked pathways generated by the standard hypergeometric method.

**Figure 2:**
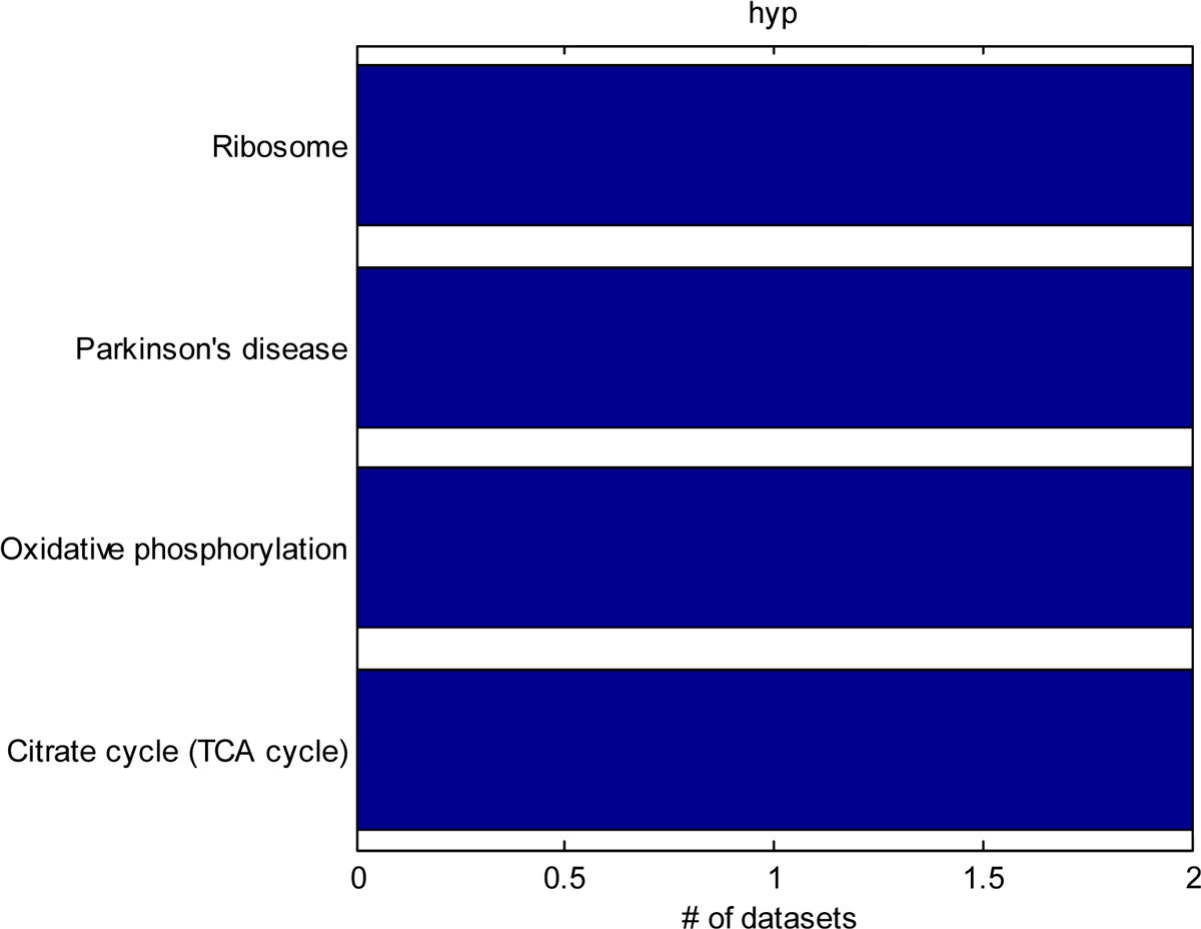
A compilation of the pathways selected by hypergeometric enrichment from the rodent neuropathic pain model datasets. Only pathways enriched in 2 or more datasets are shown.

**Figure 3:**
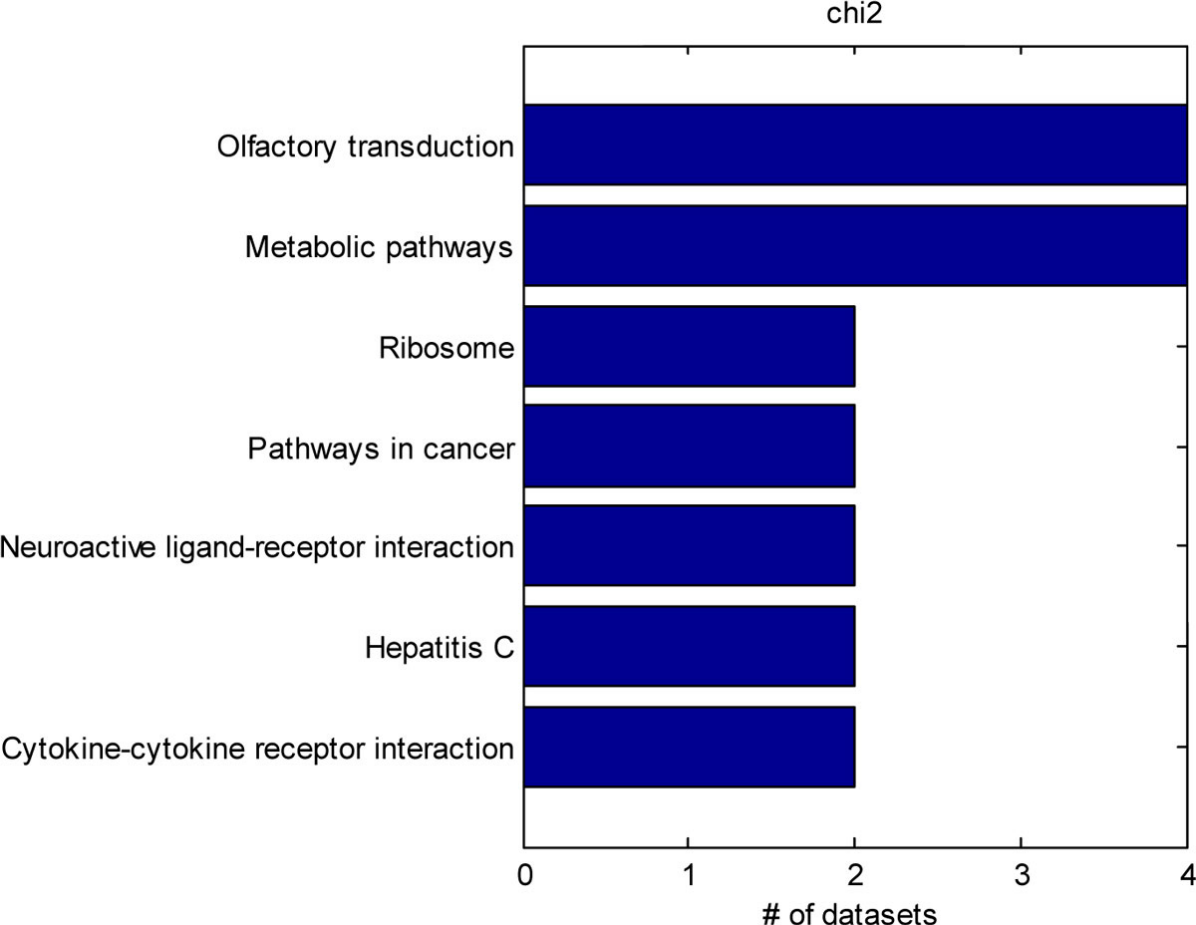
A compilation of the pathways selected by chi-squared enrichment from the rodent neuropathic pain model datasets. Only pathways enriched in 2 or more datasets are shown.

**Figure 4:**
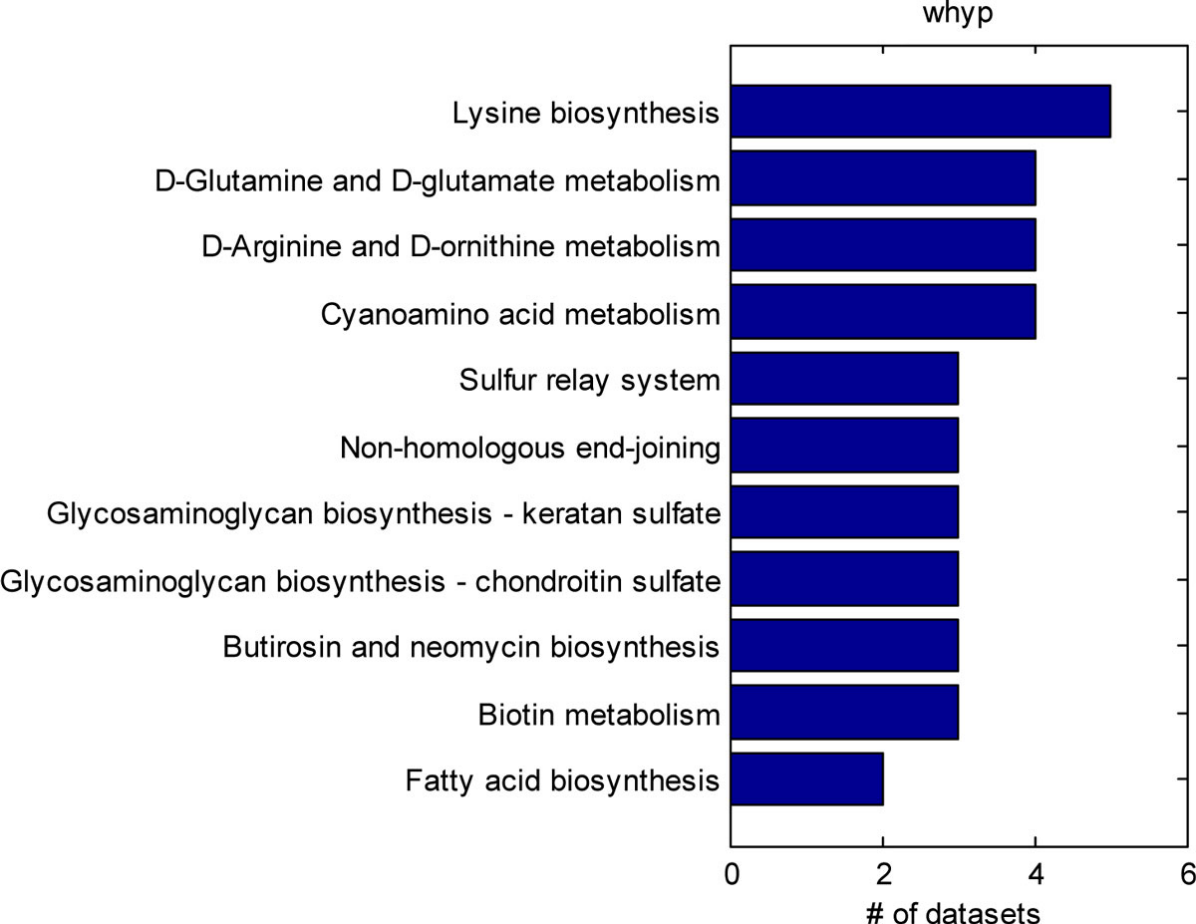
A compilation of the pathways selected by weighted hypergeometric enrichment from the rodent neuropathic pain model datasets. Only pathways enriched in 2 or more datasets are shown.

**Figure 5:**
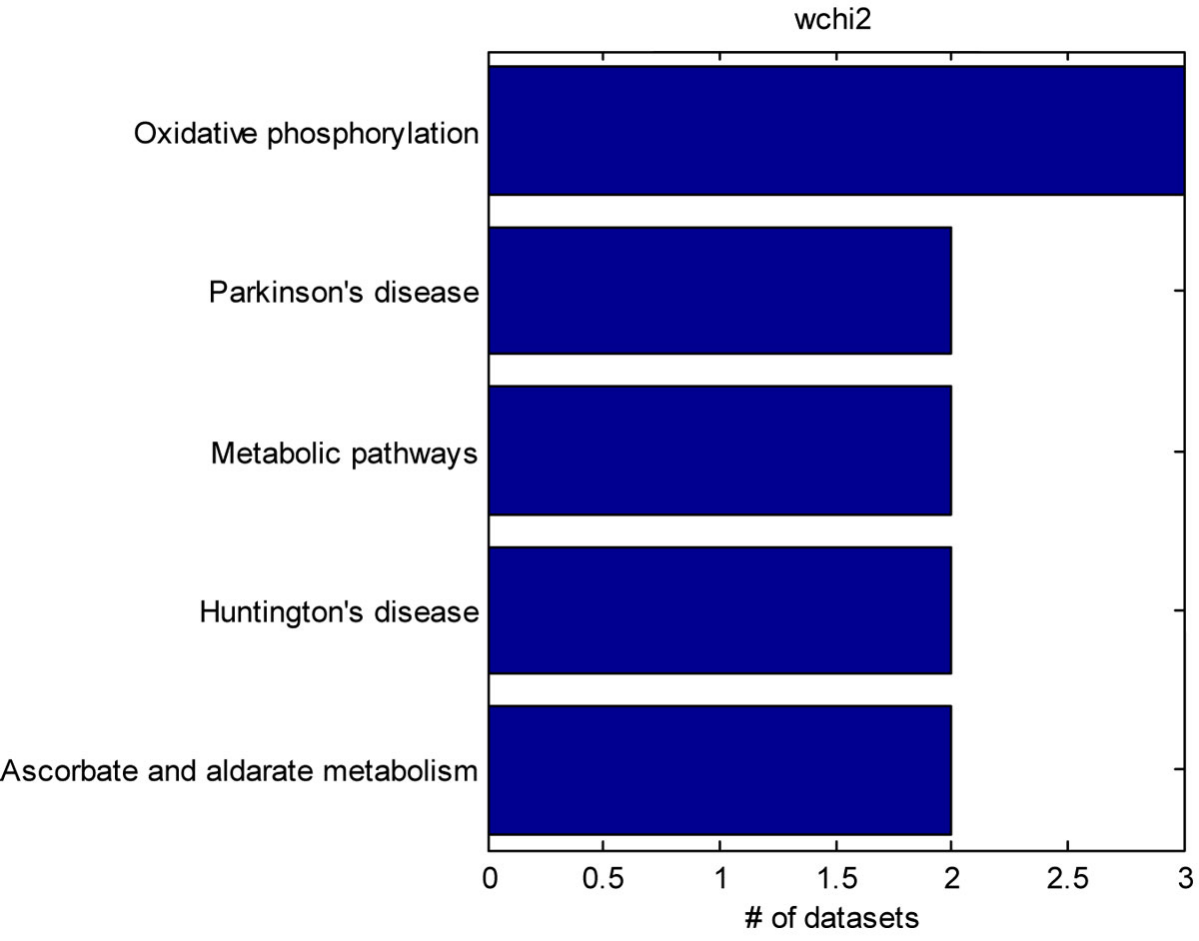
A compilation of the pathways selected by weighted chi-squared enrichment from the rodent neuropathic pain model datasets. Only pathways enriched in 2 or more datasets are shown.

We further identified 4 datasets from the Gene Expression Omnibus pertaining to rodent models of neuropathic pain. The datasets included studies utilizing spinal nerve ligation, sciatic nerve ligation, chronic constriction injury, and spared nerve injury neuropathic pain models [25,26,34,35]. Although only 4 studies were utilized, some studies contained multiple neuropathic pain models, so we examined differential gene expression across 5 different conditions. Hypergeometric, chi-squared, weighted hypergeometric, and weighted chi-squared enrichment were applied to each of the dataset. The top-10 most significantly enriched pathways were considered. The common pathways identified by each method in each of the datasets were tabulated. A power of 1 was used for the weighted enrichments, and a p-value cut-off of 0.01 was used for the unweighted enrichment. Figure 2 shows all of the KEGG pathways that were detected by hypergeometric enrichment in at least 2 datasets. Only, the ribosome, Parkinson's disease, oxidative phosphorylation, and TCA cycle pathways were identified in 2 different datasets. Figure 3 shows the KEGG pathways identified in at least 2 or more datasets by the chi-squared test. Only the Parkinson's disease and oxidative phosphorylation pathways were identified by both chi-squared and hypergeometric enrichment. Figure 4 shows the results of applying weighted hypergeometric enrichment with a power of 1. Weighted hypergeometric enrichment is able to detect pathways most consistently. The lysine biosynthesis pathway is significantly enriched in all datasets. Unlike the other enrichment methods, weighted hypergeometric enrichment identified completely different pathways consisting mainly of metabolic pathways, and pathways relating to amino acids. Figure 5 contains the results of weighted chi-squared enrichment. Parkinson's disease and oxidative phosphorylation are both consistently enriched by the weighted chi-squared method.

**Figure 6:**
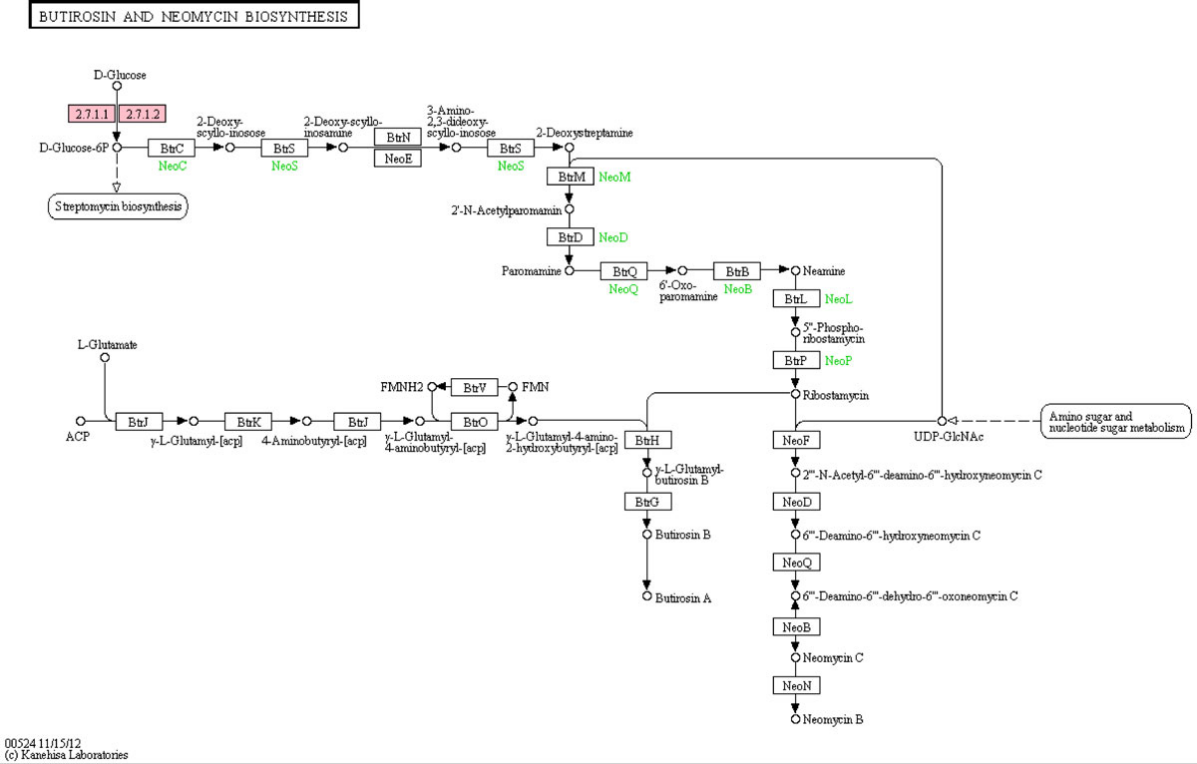
The butirosin and neomycin biosynthesis pathway. The nodes identified in the datasets are colored in pink. The node 2.7.11 corresponds to 3 different rat genes that were identified as significant.

Weighted hypergeometric enrichment detected the butirosin and neomycin biosynthesis pathway in 3 datasets, which is shown in Figure 6. This pathway was not enriched by any of the other methods in 2 or more datasets. There is evidence for action by neomycin on the nervous system; it can block the capsaicin response of rat dorsal root ganglion neurons and can block N-type and P-type voltage dependent calcium channels [36]. Neomycin may also be a transient receptor ion channel 1 (TRPV1) antagonist. TRPV1 is a ligand-gated cation channel involved in multiple pain sensation mechanisms, as a result neomcycin can alleviate pain responses [37]. Fatty acid biosynthesis is enriched in two datasets, and fatty acid metabolites can induce pain by stimulating the transient receptor potential A1 channel (TRPA1) [38]. The fact that these two pathways, which have been previously associated with neuropathic pain, are only identified by weighted hypergeometric enrichment demonstrates the potential advantage of weighted hypergeometric enrichment in identifying relevant pathways missed by the standard methods.

## Discussion

Weighted hypergeometric and chi-squared enrichment extend over-representation analysis to include change in expression of the genes and include all genes instead of a pre-selected list. These approaches enable every gene to contribute to the enrichment in proportion to their fold-change. Changing the power parameter enables one to adjust how much the expression change of the genes contributes to the enrichment score of the pathway. Our approach combines ORA and FCS methodologies. Unlike GSEA [15] our methods can detect pathways comprised of both up and down regulated genes by means of the score calculated for each gene. This is because we consider only the magnitude of expression change and not its direction with our score. There already exists a modification of GSEA that allows enrichment of pathways with bidirectional gene expression [39].

Weighted enrichment methods are much more conservative than unweighted methods. Because the weighted hypergeometric enrichment methods are so conservative, they produce no significant results when corrected for multiple comparisons. The Benjamini-Hochberg false discovery correction [40] was applied to the weighted enrichment, and the results are depicted in Tables 2, 3, 4. Table 2 shows that after false discovery rate (FDR) correction there are no significant pathways (p < 0.05), and that each pathway has an FDR-corrected p-value of 1. However, the FDR correction is not suitable for application to enrichment analysis because FDR has a high variability and should be applied to a larger number of p-values than those generated by enrichment [41]. Furthermore, it has been shown that most multiple comparison corrections decrease the power of the analysis and are also too conservative [13,42]. Additionally, the p-values resulting from enrichment analyses can be fragile and sensitive to non-statistical aspects of their calculation such as the data sources or the mapping of gene names between different conventions; these issues cannot be resolved by correction for multiple comparisons [13]. Huang et al. advise using prior biological knowledge to assess the enriched pathways, and that the results of enrichment should only be guidelines for an investigator [13]. Thus, FDR values were only included to be thorough when describing the results of these methods. We advise considering only the top-10 pathways instead of multiple comparison correction. Furthermore, we evaluated the consistency of our methods by considering the top-10 pathways enriched in data from several similar experiments. We were able to demonstrate that weighted hypergeometric enrichment produced the most consistent results.

Validating the weighted enrichment methods has proved to be challenging because there is no ground truth to compare the enriched pathways against. As a result, validation of the methods was performed based on literature search, which is not a complete or objective analysis. Literature search-based validation is biased towards already known pathways. There is no way of knowing whether pathways enriched by the dataset that have not been previously identified in the literature are actually associated with the disease or are falsely identified as enriched. Furthermore, our method is still sensitive to the handling of gene identifier mapping. Another drawback of this methodology is that it ignores the topology of the pathways. It is possible, for example, that an increase in the expression of a gene could be canceled out by a decrease in the expression of a downstream gene that is up regulated by the first gene. There is no way to address this situation when using our method. However, this drawback does not apply to the enrichment of Gene Ontology terms, which are arranged hierarchically.

We have proposed weighted hypergeometric and chi-squared methods to enrich gene sets. These methods can produce more biologically relevant results for KEGG pathway enrichment than the standard hypergeometric approach, despite the fact that the problem of Type II errors is inadequately addressed by correcting for multiple comparisons. We also showed that our method tends to produce more consistent results when using data from similar experiments. Despite only showing the results of KEGG pathway enrichment, these methods can also be applied to the Gene Ontology classifications as well as any other set of genes.

## Competing interests

The authors declare that they have no competing interests.

## Authors' contributions

AS and RQ conceived of the methodology. RQ implemented the method, and AS and RQ both contributed to the manuscript.

## Declarations

The publication costs for this article were funded by the corresponding author.

This article has been published as part of *BMC Systems Biology *Volume 7 Supplement 4, 2013: Selected articles from the IEEE International Conference on Bioinformatics and Biomedicine 2012: Systems Biology. The full contents of the supplement are available online at http://www.biomedcentral.com/bmcsystbiol/supplements/7/S4.
